# CBP/p300 and SIRT1 Are Involved in Transcriptional Regulation of S-Phase Specific Histone Genes

**DOI:** 10.1371/journal.pone.0022088

**Published:** 2011-07-15

**Authors:** Hongpeng He, Fa-Xing Yu, Chi Sun, Yan Luo

**Affiliations:** Institute of Molecular and Cell Biology, Singapore, Singapore; Texas A&M University, United States of America

## Abstract

**Background:**

Histones constitute a type of essential nuclear proteins important for chromatin structure and functions. The expression of major histones is strictly confined to the S phase of a cell cycle and tightly coupled to DNA replication.

**Methodology/Principal Findings:**

With RT-qPCR and ChIP assays, we investigated transcriptional regulation of the S-phase specific histone genes and found that the acetylation level of histones on core histone gene promoters fluctuated during cell cycle in a pattern similar to RNA polymerase II association. Further, we showed that CBP/p300 and SIRT1 were recruited to histone gene promoters in an NPAT-dependent manner, knockdown of which affected histone acetylation on histone gene promoters and histone gene transcription.

**Significance:**

These observations contribute to further understanding of the mechanism by which the expression of canonical histone genes is regulated, and also implicate a link between histone expression and DNA damage repair and cell metabolism.

## Introduction

There are two groups of histones in mammalian cells: a small group of histone variants (also dubbed replacement histones) that are constitutively expressed and a major group of canonical histones that are expressed in an S-phase specific fashion [1], [2]. The S-phase specific histones (core histones H2A, H2B, H3, H4 and linker histone H1) are indispensable for the assembly of newly synthesized DNA into chromatin in the S phase of a cell cycle. Inhibition of S-phase specific histone synthesis leads to suspension of DNA replication and arrest of cell cycle progression [3]. In addition, coordinated (and stoichiometric) expression of core histone subtypes helps to maintain genomic integrity; disrupting the stoichiometry was shown to result in genomic instability due to abnormal centromere structures and chromosome segregation [4]–[6]. Furthermore, as scaffold proteins of chromatin, histones, the N-terminal tails of core histones in particular, are frequently modified. Histone modifications have crucial effects on chromatin related processes such as transcription, DNA replication, DNA damage repair and genetic recombination [7]–[11]. Thus, proper expression of histone genes and proper epigenetic modifications of histone proteins are of fundamental importance for diverse aspects of cellular physiology.

Histone expression is regulated at both transcriptional and posttranscriptional levels [12]. Upon S-phase entry, the histone gene transcription is induced by 3- to 10-fold [13], which in conjunction with increased pre-mRNA processing (about 10-fold) contributes to up to 35-fold increase of steady-state histone mRNA levels in the S-phase [14]. Earlier studies showed that the promoters of mammalian core histone subtype genes utilize distinct subtype specific consensus sequences (SSCSs) [2], [15], [16] and that different proteins or protein complexes are associated with respective SSCS to dictate S-phase specific activation of respective genes; for example, the transcription factor HINF-P is specific for the H4 gene promoter and the transcription factor Oct-1 with its cognate co-activator OCA-S (Oct-1 co-activator in S-phase, a protein complex) is specific for the H2B gene promoter [17], [18]. Upstream of these subtype-specific transcription regulators and downstream of cyclin E/cdk2 lies NPAT , a cyclin E/cdk2 substrate and a global histone expression regulator; the phosphorylation of NPAT at the G1/S border links the cyclin E/cdk2 signaling to histone expression [19]–[24]. Importantly, cyclin E/cdk2, NPAT, all replication-dependent core histone genes and cognate transcription factors and/or co-activators are co-localized in nuclear domains dubbed Cajal Bodies [19], [23]. NPAT, which associates with different core histone promoters, may mediate the assembly of transcription machineries of all core histone genes in Cajal Bodies; this might explain the observation that, despite the involvement of distinct transcription factors and/or co-activators, the expression of core histone subtype genes is remarkably coordinated [2].

Histone modifications, the acetylation of histone N-terminal tails in particular, are correlated with the activation of gene transcription; genome-wide studies suggest that acetylated histones are spatially enriched in the promoter regions of actively transcribed genes whereas the histone acetylation level is relatively low in the promoter regions of transcriptionally inert genes [25], [26]. For periodically expressed genes, such as cell cycle regulated histone genes, a relationship between histone acetylation status and transcriptional activation is not yet defined.

Here we show that, on histone gene promoters, the acetylation levels of histone H3 N-terminal tail fluctuate during cell cycle, peaking at the G1/S transition. Transcriptional co-activators CBP/p300, of which the histone acetyl transferase (HAT) activity is stimulated by cyclin E/cdk2, associate with the core histone promoters, likely through an interaction(s) with NPAT. Inhibition of CBP/p300 reduces the histone acetylation levels on histone gene promoters in concert with down-regulation of histone gene expression. In addition, we also show that SIRT1, a member of the NAD+-dependent histone deacetylase complexes (HDACs), is associated with histone promoters as well to modulate histone expression in an opposite fashion as that of CBP/p300.

## Results

### Acetylation of histone proteins on the histone H2B gene promoter is cell cycle regulated

**Figure 1:**
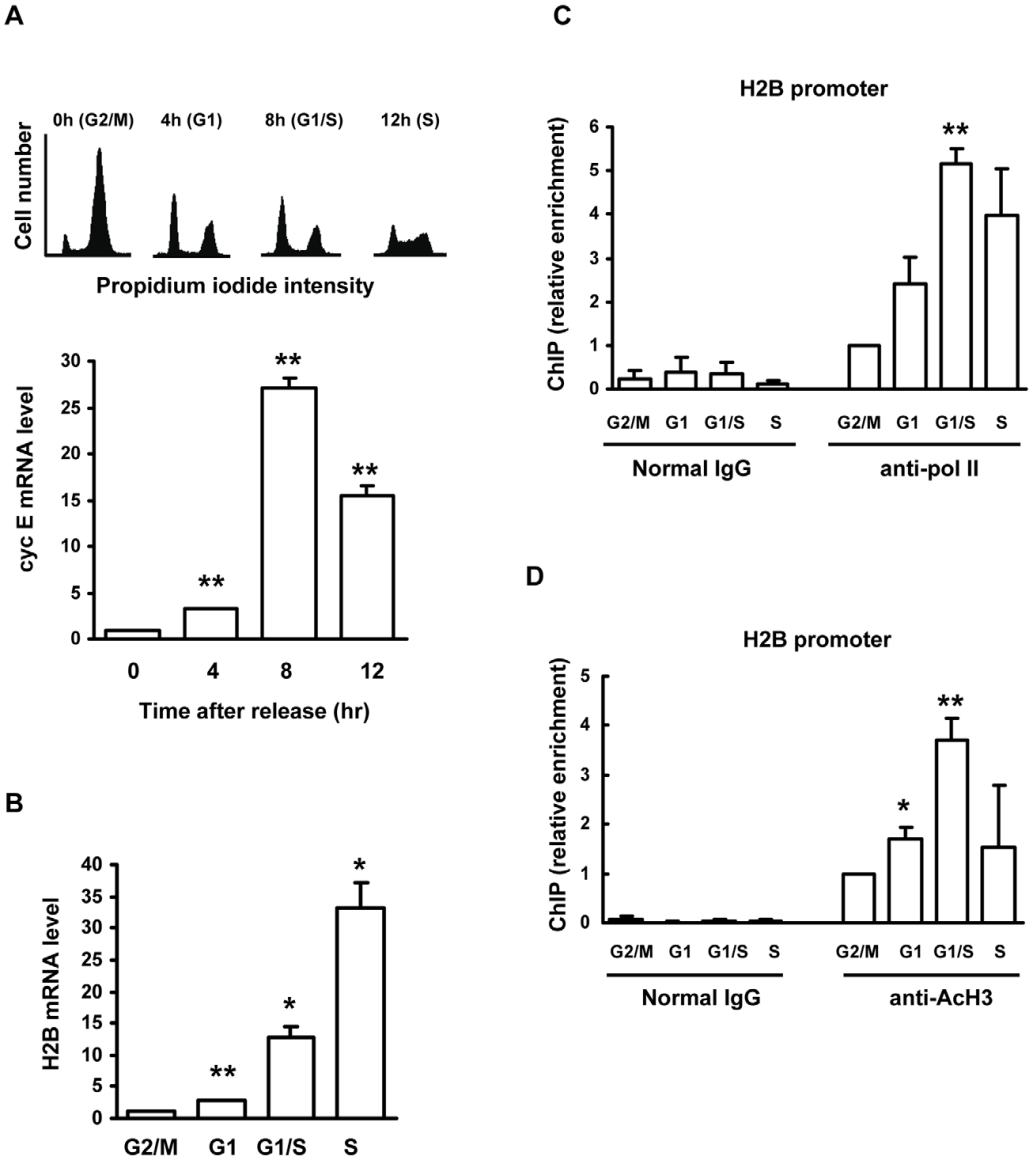
Histone H3 acetylation and RNA polymerase II association on histone promoter are cell cycle regulated. HeLa cells were treated with 75 ng/ml of nocodazole for 22 hours to arrest cells in G2/M phase. Then cells were released into cell cycle progression. At 0, 4, 8 or 12 hours after release, cells were harvested for analyses. (A) Synchronization of HeLa cells in different phases of cell cycle. FCAS was carried out to show the position of cells in the cell cycle (*upper panel*). Cyclin E mRNA level was measured with reverse transcription-quantitative real-time PCR (RT-qPCR; *lower panel*), n = 3. (B) Cell cycle regulated H2B expression. H2B mRNA level was quantified by RT-qPCR, n = 3. (C) Cell cycle regulated RNA polymerase II association with the H2B promoter. ChIP was performed with normal mouse IgG or anti-RNA polymerase II (anti-Pol II) antibodies. (D) Cell cycle regulated histone H3 acetylation on the H2B promoter. ChIP was performed with either normal rabbit IgG or anti-AcH3 antibodies. qPCR was used to quantify the enrichment of RNA polymerase II (C) or acetylated histone H3 (D) on the H2B promoter region, n = 3.

Acetylation of core histone proteins is usually taken as a marker for actively transcribed genes. To test whether the acetylation status of histone proteins on histone gene promoters changes during cell cycle progression, we synchronized HeLa cells at the G2/M phase with nocodazole and then released the cells into G1, G1/S, and S phases. Cell phases were identified with FACS (Fig. 1A, upper panel) and confirmed by expression profile of cell cycle marker, for instance the G1/S marker cyclin E (Fig. 1A, lower panel). The FACS analyses, together with the expression patterns of cyclin E (Fig. 1A), indicate that nocodazole-treated HeLa cells were at the G2/M phase before the release and reached the G1 phase, G1/S transition and mid-S phase at, respectively, 4 hour, 8 hour and 12 hour time points after the release.

With the synchronized cells, we measured the histone H2B mRNA expression levels (Fig. 1B); the H2B expression was low at the G2/M and G1 phases, increased ∼15-fold at the G1/S transition and reached >30-fold at the mid-S phase. These results are in line with the idea that H2B transcription is activated upon S phase entry and that H2B mRNA further accumulates at the mid-S phase, attributed to increased transcription along with enhanced posttranscriptional mRNA processing [2], [12]–[15]. The boosted histone gene transcription upon S-phase entry is presumably at the level of initiation, which can be measured by promoter recruitment of RNA polymerase II (RNAPII). We used ChIP assays to assess the initiation of transcription from the H2B gene and found that the recruitment of RNAPII to the H2B promoter was lowest at the G2/M phase, slightly increased in the G1 phase, peaked at the G1/S transition and started to decrease in the mid-S phase (Fig. 1C).

To explore whether the oscillatory RNAPII recruitment pattern (Fig. 1C) was correlated with certain histone acetylation status of the H2B promoter, we measured the histone H3 acetylation levels on this promoter with ChIP assays using antibody against Lys 9 and Lys 14 acetylated histone H3 (H3-K9K14Ac). The enrichment of acetylated histone H3 exhibited a pattern (Fig. 1D) similar to that of RNAPII recruitment (Fig. 1C). These results hence suggest that the up-regulated H3 acetylation on the H2B promoter is an important marker for boosted H2B transcription initiation upon S-phase entry.

### CBP and p300 are required for histone gene expression and histone promoter acetylation

There are at least 15 HATs in mammalian cells [27], of which the CBP/p300 HAT activities were previously [28] shown to be controlled by cyclin E/cdk2 that is also upstream of the histone gene transcription [19], [23]. Importantly, the CBP/p300 HAT activities were shown to be the strongest at the G1/S border [28], in which histone acetylation levels on the H2B promoter were found to peak (Fig. 1D). These findings suggest that the CBP and p300 are the potential HATs that acetylate histone proteins on histone gene promoters to activate histone expression.

**Figure 2:**
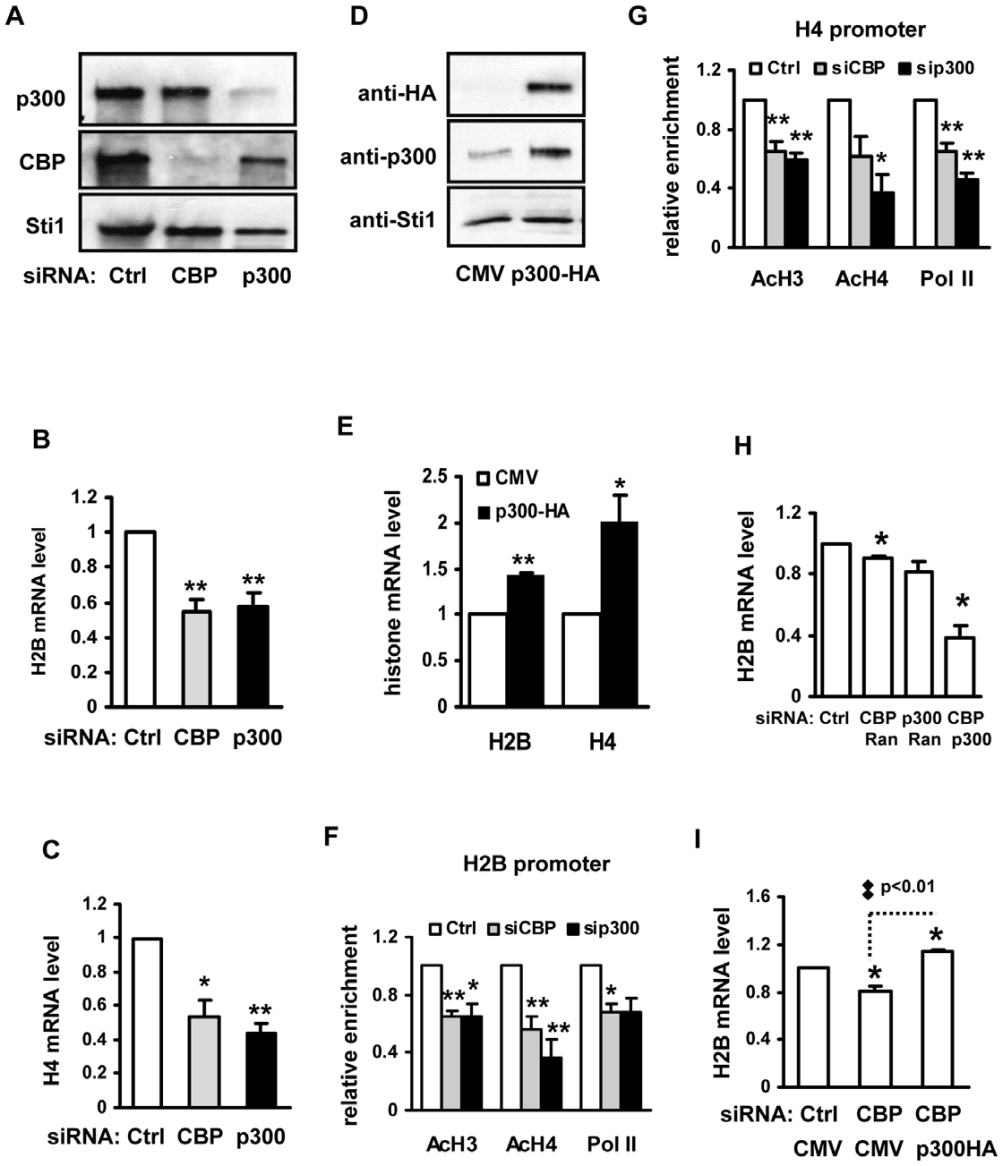
CBP and p300 are required for histone gene expression and histone acetylation on histone promoters. In A–C, F and G, HeLa cells were transfected with 100 nM of CBP or p300 specific siRNA with random siRNA as control (Ctrl). For D and E, HeLa cells were transfected with 3 µg/ml of p300-HA encoding plasmids or empty vector CMV. Cells were harvested for Western-Blot, RT-qPCR or ChIP analysis 48 hours post transfection. (A) The specificity and efficiency of the knockdown of CBP and p300. Western-Blot was performed with rabbit anti-p300, mouse anti-CBP or rabbit anti-Sti1 antibody as indicated. Sti1 (Stress-inducible protein 1) level was used as a loading control. (B) Decreased H2B expression in CBP or p300 knockdown cells. n = 5. (C) Decreased H4 expression in CBP or p300 knockdown cells. n = 5. (D) Over-expression of HA-tagged p300. (E) Increased H2B and H4 expression in p300 over-expressing cells. n = 3. (F) Reduced level of acetylated H3, H4 and RNAPII at the H2B promoter by CBP or p300 knockdown. n = 5. (G) Reduced level of acetylated H3, H4 and RNAPII at the H4 promoter by CBP or p300 knockdown. n = 4. (H) The additive effect of double knockdown of CBP and p300. HeLa cells were transfected with 50 nM of CBP and/or p300 specific siRNA; compensating dosage of random siRNA was used to make the concentration of total siRNA at 100 nM, n = 3. (I) Compensation of reduced H2B expression resulted from CBP knockdown by p300 over-expression. Comparison between column 2 and 3 was analyzed with unpaired *t* test. n = 3.

To test whether CBP and its homolog p300 are involved in histone gene activation, we knocked down CBP or p300 expression with siRNAs. The decrease of CBP and p300 protein levels was examined with Western-Blot, which exhibited relatively complete knockdowns and reinforced the specificity of respective siRNAs and antibodies (Fig. 2A). The expression of core histone genes is normally coordinated [2], [15], and we chose H2B and H4 genes as examples. As shown in Figure 2B and 2C, siRNA-mediated knockdown of CBP or p300 reduced the expression of H2B and H4 genes by 40–60%. These results suggest that transcriptional co-activators CBP and p300 are involved in activation of histone expression, a notion reinforced by an experiment with p300 over-expression in HeLa cells shown to enhance H2B and H4 expression up to 1.4 and 1.8 fold, respectively (Fig. 2D and 2E).

To ask whether CBP and p300 regulate histone genes via acetylating histones on the histone gene promoters, we used ChIP assays to assess the histone acetylation status of H2B and H4 promoters in HeLa cells in which the expression of CBP or p300 was knocked down. In CBP- or p300-specific siRNA transfected cells, H3 and H4 acetylation levels at, as well as RNAPII recruitment to, the H2B and H4 promoters were reduced by 35–60% (Fig. 2F and 2G). Thus, CBP and p300 are involved in acetylating histones at the histone gene promoters, which would help to configure chromatin structure to facilitate RNAPII recruitment.

CBP and its homolog p300 are usually identical in functions as transcriptional co-activators [27], [29]–[31]; however, there had been reports that they have distinct transcriptional functions for certain genes at different development stages [32], [33]. To test whether CBP and p300 are functionally equivalent in the regulation of histone expression, we compared the effects of double knockdown of CBP and p300 with that of single knockdown. In these double knockdown experiments, the total dosage of siRNAs was the same as that in single knockdowns, i.e., the concentration of siRNA for CBP or p300 was reduced in half. As shown in Figure 2H, single knockdown of CBP or p300 with the mixture of random siRNA and specific siRNA resulted in slightly decrease of H2B mRNA level by 10–20%. Whereas, double knockdown of CBP and p300 generated a much more dramatic decrease (about 60%) in histone H2B mRNA level than single knockdown, indicating an additive effect. Meanwhile, in HeLa cells with a CBP knockdown achieved with co-transfection of CBP specific siRNA and CMV control plasmids, histone H2B mRNA level decreased by 20% which was compensated by CMV-driven over-expression of p300 (Fig. 2I). Taken together, the results suggest a functional redundancy of p300 and CBP as far as histone expression is concerned.

**Figure 3:**
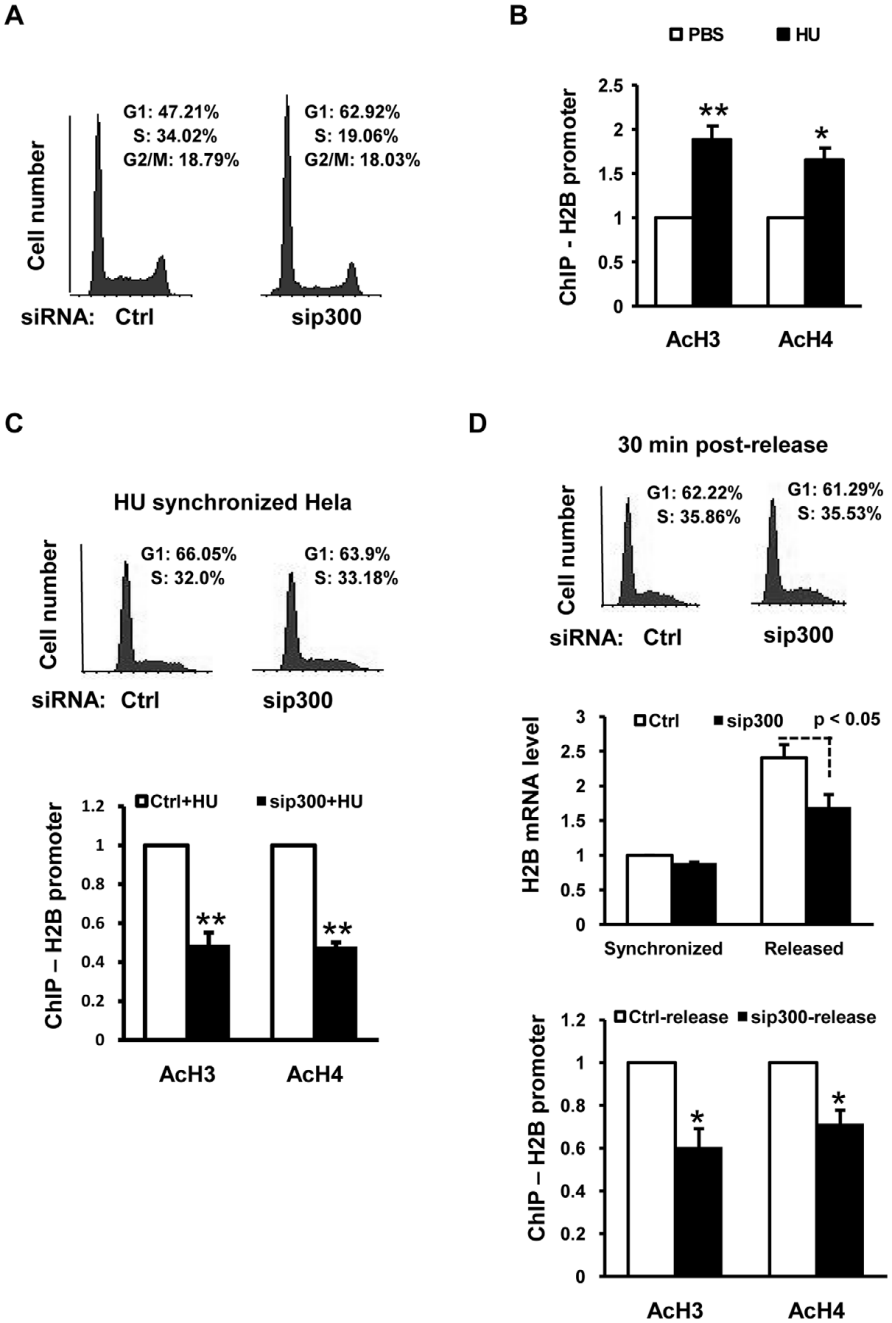
The acetylation levels of histones at histone gene promoters decrease in cells with p300 knockdown. (A) p300 downregulation by siRNA resulted in cell accumulation in G1 phase. siRNA transfection was as described in Fig. 2A. (B) Acetylated histone H3 and H4 on the H2B promoter increased in HU treated cells. n = 4. HeLa cells were treated with 2.5 mM of HU for 24 hours before being harvested. HU was dissolved in PBS buffer. (C) Histone H2B promoter acetylation decreased in HU-synchronized cells with p300 knockdown. HeLa cells were transfected with sip300 or control siRNA as indicated. 24 hours after transfection, cells were switched to fresh media with 2.5 mM of HU for another 24 hours. n = 3. (D) Histone H2B mRNA level and histone promoter acetylation level were reduced in p300 knockdown cells in early S phase. Cells synchronized as described in (C) were released into S-phase for 30 minutes before being harvested for FACS, RT-qPCR and ChIP. Comparison of H2B mRNA levels between released control and sip300 groups was analyzed with unpaired *t* test. n = 4.

CBP/p300 has been reported to be required for cells to enter S-phase [34]. Indeed, our FACS analysis showed increased percentages of G1-phase cells and decreased percentages of S-phase cells in p300-knockdown cells (Fig. 3A). Given that histone expression is tightly coupled to DNA replication [1], [12], it is reasonable to argue that the histone expression down-regulation in CBP/p300 knockdown cells (Fig. 2B and 2C) is solely due to a feedback mechanism from DNA replication deficiency rather than primary effects of CBP/p300 knockdown on histone genes.

To resolve this issue, we investigated the effects of CBP/p300 knockdown in synchronized cells, which normally retain prominent levels of histone acetylation on histone promoters (i.e., at G1/S border, see Fig. 1D). HU, which inhibits DNA replication by blocking the formation of dNTPs [35], is able to arrest cells at the G1/S border and early S phase [3]. ChIP with HU synchronized cells showed that histone H3 and H4 acetylation on histone H2B promoter was not only manifest but also increased by 1.7–2 fold as compared with random cells (Fig. 3B). We employed HU to synchronize cells that were transfected with control or p300-specific siRNA. Before release, the overall G1 and S-phase partitions did not vary significantly between the two samples (Fig. 3C, upper panel). At this point, ChIP assays showed that histone H3 and H4 acetylation levels on H2B promoter were much reduced in p300 knockdown cells (Fig. 3C, lower panel). HU-synchronized cells generally exhibit low level of histone expression [3], which presumably reflects a feed-back mechanism from suppressed DNA replication to histone expression. After we released the HU-synchronized cells for 30 minutes, the H2B expression was significantly boosted (Fig. 3D, middle panel); however, the levels in sip300 transfected cells were statistically lower as compared with the control cells (Fig. 3D, middle panel) while the cell cycle profiles of the two samples remained insignificantly different (Fig. 3D, upper panel). Consistently, the acetylation level of histone H2B promoter in p300 knockdown cells remained significantly lower than that in control cells (Fig. 3D, bottom panel). We therefore conclude that reduced histone expression in CBP/p300 deficient cells is most-likely a result of reduced histone acetylation on histone gene promoters, which is in agreement with the data in Figure 2, and is due to primary effects of CBP/p300 knockdown on histone genes.

### CBP and p300 associate with histone gene promoters in an NPAT-dependent manner

**Figure 4:**
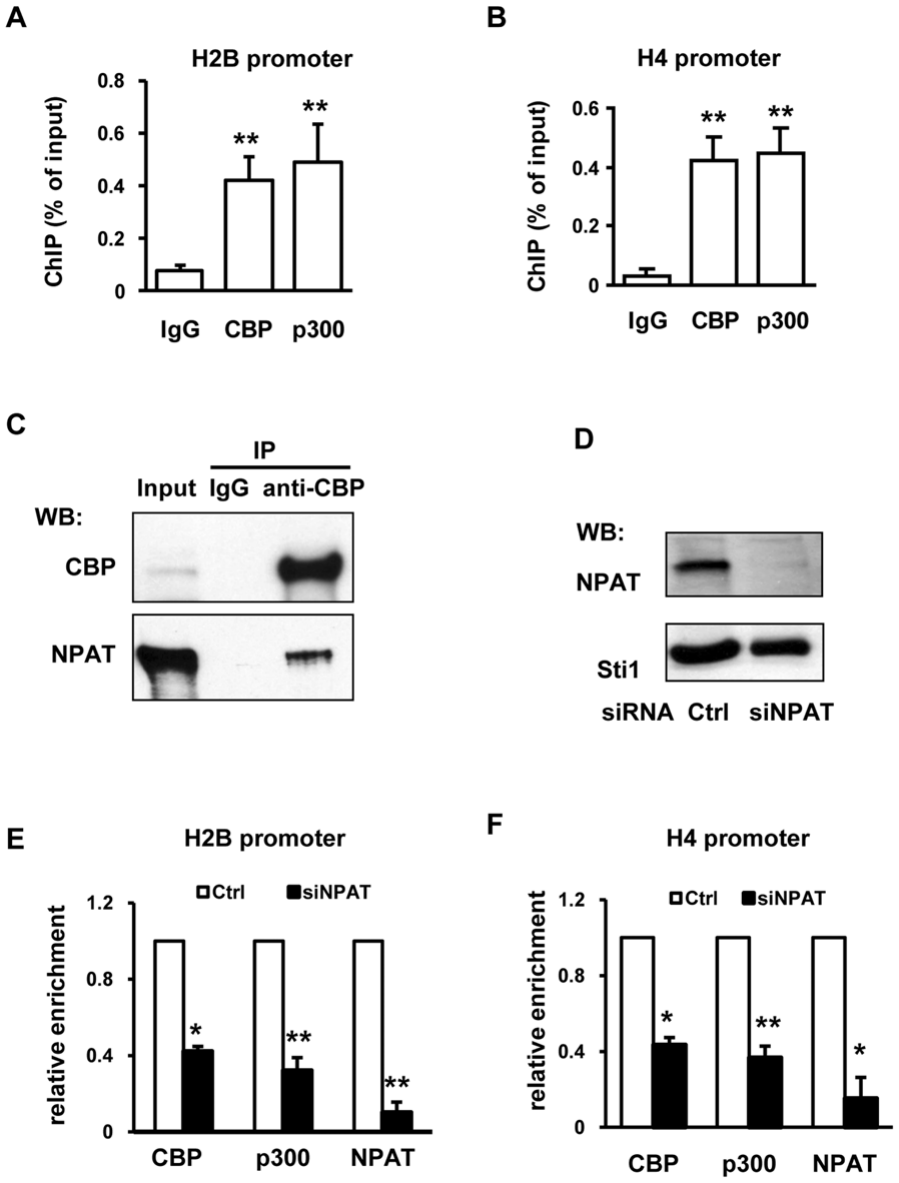
CBP and p300 associate with histone promoters in an NPAT-dependent manner. ChIP assays were performed with rabbit anti-CBP or anti-p300 antibodies. (A) CBP and p300 association with the H2B promoter. n = 5. (B) CBP and p300 association with the H4 promoter. n = 5. (C) Co-immunoprecipitation of CBP with NPAT. Whole cell extracts of HeLa cells transfected with pCMV-NPAT were used to carry out co-immunoprecipitation with rabbit anti-CBP antibodies. Precipitated proteins were detected with Western-blot using mouse anti-CBP antibodies or mouse anti-NPAT antibodies as indicated. (D) The efficacy of NPAT knockdown. Western-Blot was performed with rabbit anti-NPAT or rabbit anti-Sti1 antibody as indicated. Sti1 was used as a loading control. (E) Reduced association of CBP and p300 with the H2B promoter in NPAT knockdown cells. n = 3. (F) Reduced association of CBP and p300 with the H4 promoter in NPAT knockdown cells, n = 3.

To examine whether CBP/p300 regulate histone promoter activity, we performed ChIP assays with CBP or p300 antibodies. As shown in Figure 4A and 4B, both CBP and p300 associated with histone promoters, supporting that these transcriptional co-activators play a direct role in histone gene regulation. Since CBP and p300 do not directly bind DNA, their association with histone gene promoters should be mediated by other transcriptional regulators. The global histone expression regulator NPAT was shown to co-localize with CBP at the G1/S transition and interact with CBP [36]. Thus, we proposed that NPAT played role(s) in CBP/p300 recruitment to histone gene promoters, presumably via CBP/p300-NPAT interaction(s). Hence, we performed a coimmunoprecipitation assay with anti-CBP antibodies, and results showed co-precipitation of NPAT with CBP (Fig. 4C). To test whether NPAT is needed for CBP and p300 recruitment to histone promoters, we knocked down the NPAT expression in HeLa cells (Fig. 4D) and then performed ChIP assays, which revealed 60–70% reduced CBP and p300 associations with histone promoters (Fig. 4E and 4F). These results suggest that the CBP and p300 associations with histone promoters are NPAT-dependent.

**Figure 5:**
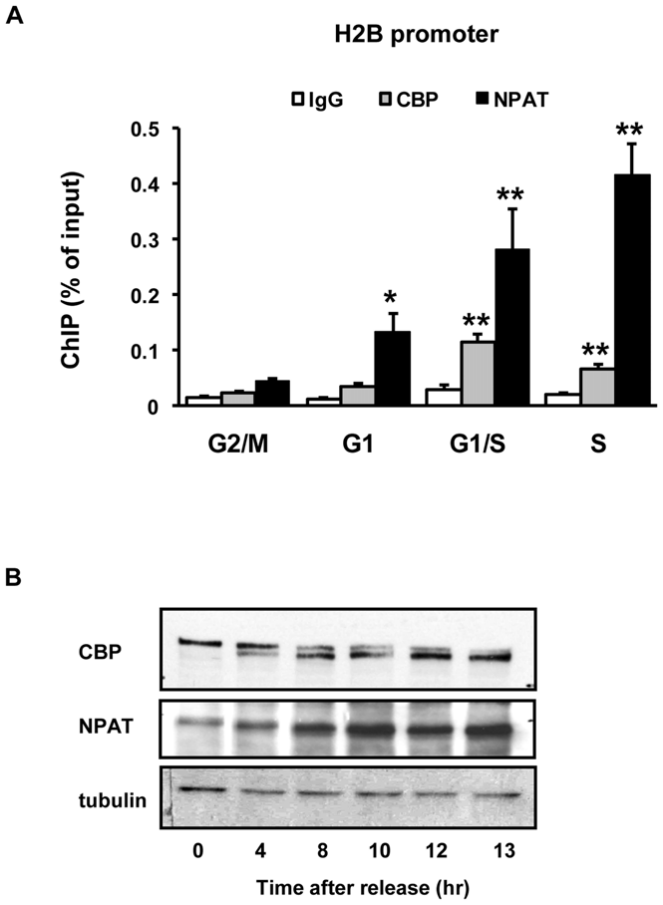
Cell cycle regulated association of CBP and NPAT with the H2B promoter. (A) CBP and NPAT association with histone promoter in different phases of a cell cycle. HeLa cells were synchronized at G2/M, G1, G1/S and mid-S phases as described in Figure 1. ChIP assays were performed with rabbit anti-CBP or rabbit anti-NPAT antibodies with normal rabbit IgG as control. Enrichment of CBP or NPAT on H2B promoter at G1, G1/S or S phase was compared with that of G2/M phase, respectively, n = 5. (B) CBP and NPAT protein levels in different phases of a cell cycle. HeLa cells were synchronized with nocodazole and then released for different time points as indicated. Whole cells extracts were used for Western-Blot analyses. Membranes were blotted with mouse anti-CBP, rabbit anti-NPAT or mouse anti-tubulin. Tubulin was used as loading control.

NPAT association with histone promoters is cell cycle regulated [17], [19]. Therefore, we proposed that the anchorage of CBP/p300 to histone promoters was also cell cycle regulated if CBP and p300 are genuinely recruited to histone promoters in an NPAT-dependent fashion. Indeed, ChIP assays with nocodazole synchronized HeLa cells showed that CBP association with the H2B promoter was low at G2/M and G1 phases, significantly increased at G1/S phase but started to decrease in mid-S phase (Fig. 5A). Interestingly, the oscillated association of CBP mimics the pattern of histone H3 acetylation on the H2B promoter (Fig. 1D).

It bears mention that, although the association of CBP with H2B promoter decreased in mid-S phase as compared with the G1/S transition, NPAT enrichment on histone H2B promoter further increased in mid-S phase (Fig. 5A). It was shown by others that NPAT expression is cell cycle regulated [37], which might explain the increased association of NPAT with histone promoters in S-phase. We thus examined the protein levels of CBP and NPAT in cells released from nocodazole block. As shown in Figure 5B, NPAT protein level increased gradually after release and remained at high level in mid-S phase; whereas, the total protein level of CBP kept constant through the cell cycle with an obvious shift from a higher mobility band in G2/M phase to a lower mobility band in mid-S phase. This mobility shift might results from cell cycle regulated modification(s) of CBP, given that CBP was previously shown to be targeted by various protein kinases [28], [38], [39]. Therefore, we propose that the decreased association of CBP after mid-S phase may result from the change of protein modification status. Alternatively, there might be yet another “bridging” regulator that potentially mediates a CBP-NPAT interaction, the decrease in the level or activity of this factor in mid-S phase might prompt the decreased recruitment of CBP. Further increased NPAT recruitment at mid-S phase may have certain (other) physiological function(s), e.g., mediating the co-repressor(s) recruitment that facilitates the histone expression declination typical of late S-phase (see below and Discussion).

### The CBP/p300 HAT activity is important for histone gene transcription

CBP and p300 are large proteins with multiple domains, including a HAT domain, a Bromo-domain, a TFIIB binding domain and a few of other protein binding domains [27], [33], [40]. The HAT domain is not always indispensible for their transcription co-factor function; for instance, sometimes CBP and p300 act, independent of HAT activities, as a bridging factor facilitating RNAPII recruitment [41], [42].

**Figure 6:**
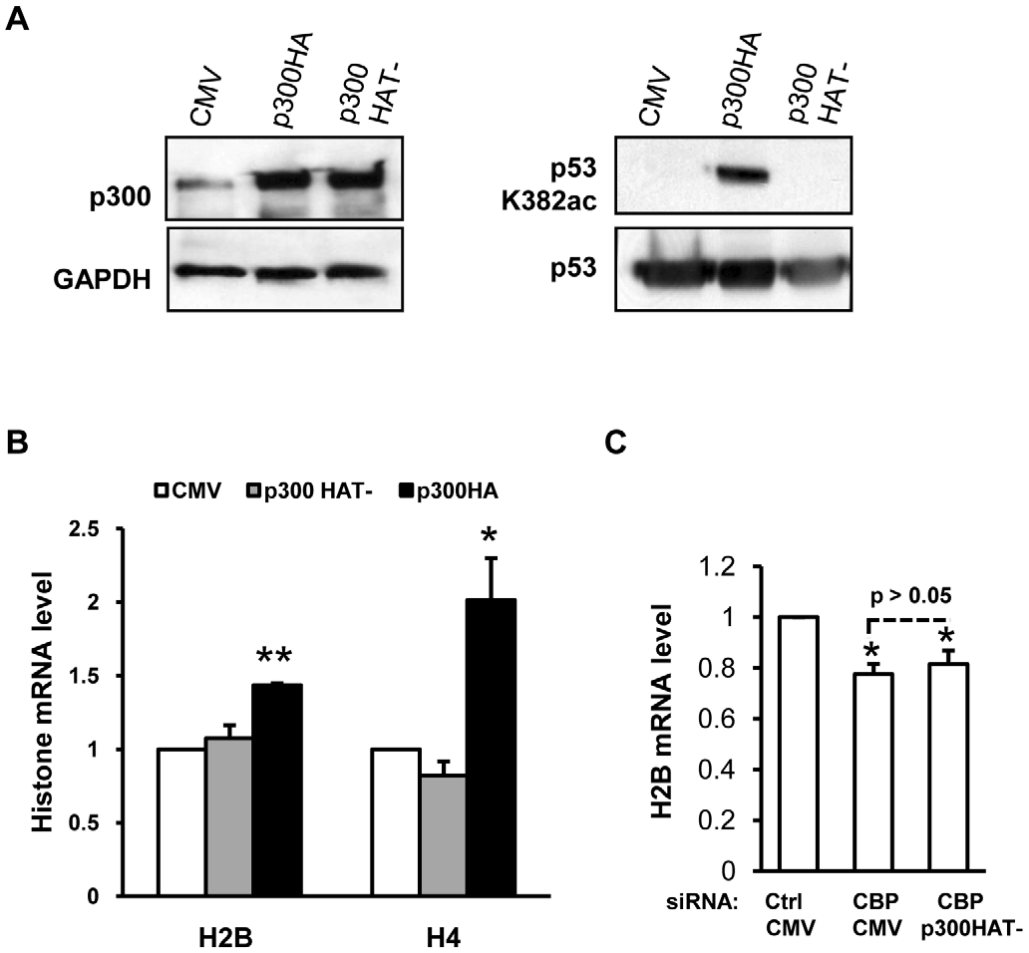
The HAT activity of CBP/p300 is important for histone gene transcription. (A) HAT activity of ectopically expressed p300. Plasmids pCMV-p300HA which encodes HA-tagged p300 or pcDNA3.1-p300HAT- which encodes p300 (HAT-) mutant were transfected into HeLa (left panels) or 293T cells (right panels). Western-blot was employed to determine protein expression and the HAT activity. (B) Histone H2B and H4 transcription was enhanced by p300HA but not p300 (HAT-). n = 4. (C) p300 (HAT-) mutant was unable to rescue the down-regulation of H2B transcription resulted from CBP knockdown. HeLa cells were co-transfected with siRNA and plasmid DNA as indicated. 48 hours after transfection, cells were harvested for RT-real time qPCR. Comparison between column 2 and 3 was analyzed with unpaired *t* test. n = 4.

To test whether CBP/p300 HAT activity is required for activating histone gene expression, we introduced p300 with or without point mutations in HAT domain [43], [44] into HeLa cells (Fig. 6A, left panels). In addition to histones, CBP/p300 was shown to acetylate lysine residue K382 of p53 [45]. We employed HEK293T cells, known to express relatively high level of p53 thus for easier detection, and confirmed the lysine acetyltransferase activities for the HA-tagged p300 and lack of the activity for the p300 (HAT-) mutant (Fig. 6A, right panels). In HeLa cells ectopically expressing the HA-tagged p300, histone mRNA levels increased whereas histone H2B and H4 expression was not changed in p300 (HAT-) expressing cells (Fig. 6B). In Figure 2I, the HA-tagged p300 was demonstrated to be able to rescue H2B gene down-regulation caused by CBP knockdown. Here, we measured H2B mRNA level in HeLa cells co-transfected with siCBP and p300 (HAT-) encoding plasmids and found that p300 (HAT-) failed to compensate for the loss of CBP in histone gene expression (Fig. 6C). Hence, the HAT activity of CBP/p300 is important for histone gene transcription.

### SIRT1 is involved in the regulation of histone gene promoters

Given the dynamic nature of histone acetylation, we next asked whether a reversed process might be employed to deacetylate histones on histone promoters to fine-tune histone expression during S-phase progression, and, if so, we were interested in identifying involved HDAC(s).

**Figure 7:**
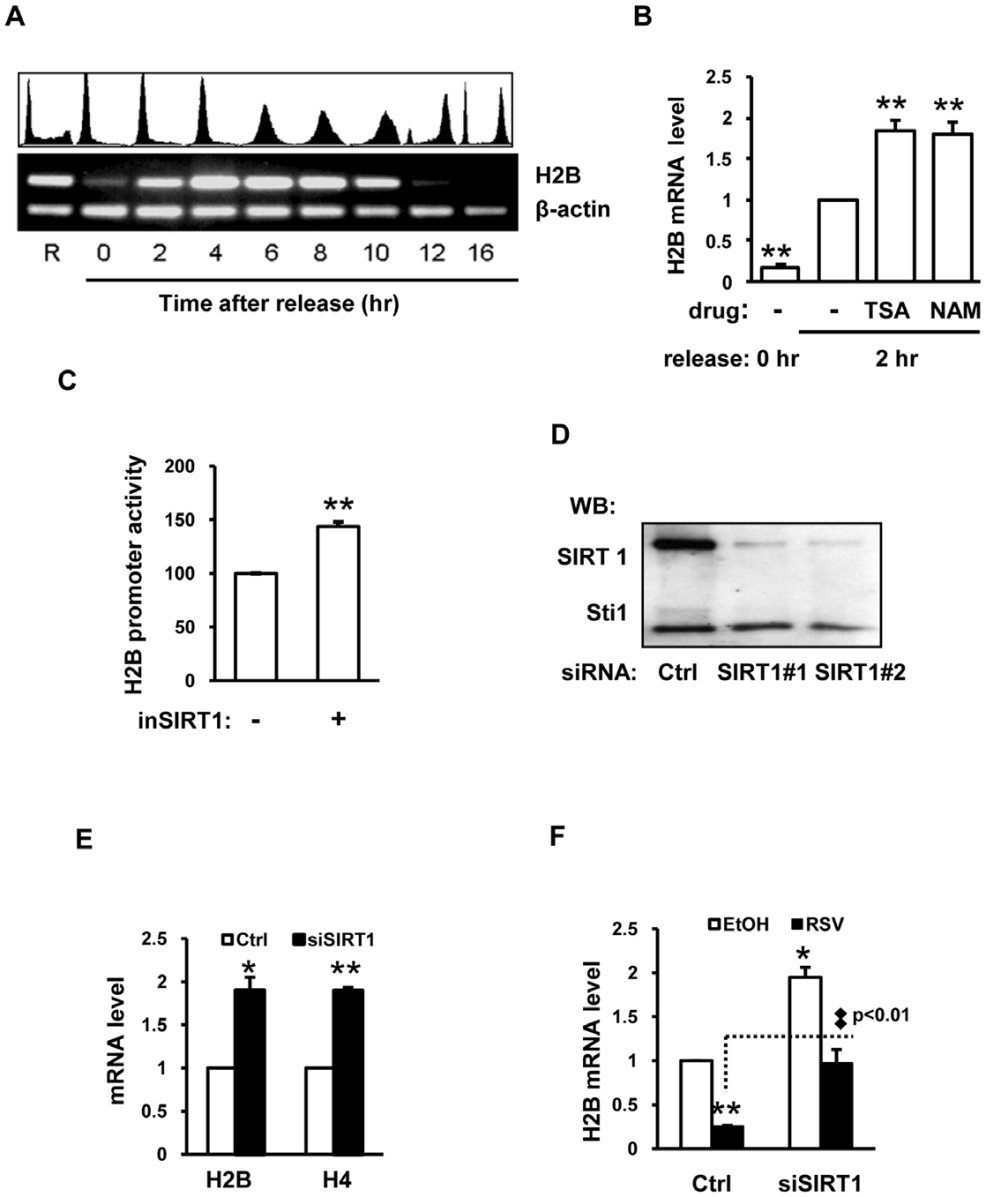
Involvement of SIRT1 in regulating the expression of S-phase specific histone genes. (A) H2B expression pattern of HeLa cells released from HU treatment. HeLa cells were synchronized at G1/S border with HU and then released into S phase. Cells were harvested at 2 hour interval and were analyzed with FACS and RT-PCR. 0 hour, G1/S border; 2 hours, early S-phase; 4 and 6 hours, mid-S-phase; 8 and 10 hours, late S-phase; 12 hours, S/G2 border; 16 hour, G2/M phase. (B) Enhanced H2B expression by HDAC inhibitors. HeLa cells synchronized at G1/S transition with HU were released and concomitantly treated with 2 µM TSA or 40 mM NAM for 2 hours. H2B mRNA level at 2 hours post release was set as 1, n = 4. (C) Increased H2B promoter activity as a result of inhibiting SIRT1. n = 3. (D) The efficacy of SIRT1 knockdown. Western-Blot was used to determine the SIRT1 protein level. The level of Sti1 serves as a control. (E) Up-regulated H2B and H4 expression in SIRT1 knockdown cells, n = 3. (F) Repressed H2B expression upon SIRT1 activation. HeLa cells were transfected with SIRT1 specific siRNA for 45 hours and then treated with 20 µM of resveratrol for additional 3 hours, n = 3. RSV, resveratrol, was dissolved in ethanol. Comparison between RSV treated groups with or without siSIRT1 transfection was analyzed with unpaired *t* test.

We first studied histone expression patterns during S-phase progression. We used DNA replication blocker, HU, which can synchronize cells at the G1/S border and early S-phase (Fig. 7A); the synchronized HeLa cells were then released to progress through the S-phase and into the G2/M phase. Figure 7A exemplifies cell cycle and histone expression patterns with HU-treated/released cells (at 2 hour intervals) with expression profiles of the H2B gene as a typical example. At the G1/S border, H2B mRNA level was low presumably because of blocking of DNA replication; however, the transcriptional activation along with enhanced mRNA stability ensured markedly increased H2B expression through the early-, mid- and late-S-phases (Fig. 7A). The H2B expression sharply declined at the S/G2 border and was undetectable at the G2/M phase (Fig. 7A). Based on the above observation, we chose 0- and 2-hour time points (G1/S border to early S-phase) to investigate the potential roles of HDAC(s) in regulating histone expression.

Among three classes of HDACs in mammalian cells, class I and II HDACs are NAD+-independent, whereas class III HDACs are NAD+-dependent [46], [47]. In HeLa cells that were HU-treated/released 2 hour into the S-phase without or with TSA (an inhibitor of class I and II HDACs) or NAM (an inhibitor of class III HDACs), H2B expression was further stimulated for about 2-fold by both chemicals (Fig. 7B, compare column 3 and 4 with column 2). These findings suggest an involvement of more than one class of HDACs; however, given that histone expression is sensitive to the NAD(H) redox status [17], [48], [49], we chose to further study the role(s) of NAD+-dependent HDACs.

There are seven members of class III HDACs; only SIRT1 and SIRT6 are consistently localized in the nucleus [50], [51]. SIRT1 and p300 have been frequently found to antagonistically modify the same targets such as p53 [52]–[55] and histone H3K56 [30]. Given that CBP and p300 were already found to acetylate histones on histone gene promoters (Fig. 2), we reasoned that SIRT1 was able to regulate histone expression in an opposite manner. To prove this point, we treated HeLa cells with the SIRT1 inhibitor III that selectively inhibits the SIRT1 HDAC activity at low concentration (IC_50_ = 98 nM and 19.6 µM for SIRT1 and SIRT2, respectively, according to the description of the manufacturer). The H2B promoter activity was increased by 1.5 fold after a 24-hour treatment with 50 nM of SIRT1 inhibitor III (Fig. 7C). Therefore, SIRT1 could be the potential HDAC regulating histone genes. To confirm that SIRT1 functions as a regulator of histone genes, the SIRT1 expression was knocked down with a SIRT1-specific siRNA (Fig. 7D), which was found to be accompanied by about 2-fold increased H2B and H4 mRNA levels (Fig. 7E).

We also tested the effect of resveratrol, a SIRT1 activator, on histone expression; and the H2B mRNA level was found to decrease by 75% in resveratrol treated cells (Fig. 7F). Given that resveratrol is a chemical with multiple functions, we carried out the resveratrol treatment in SIRT1 knockdown cells as well to make sure that the effect of resveratrol on H2B expression was mediated by SIRT1 activation, and found that the SIRT1 knockdown was able to partially reverse the inhibitory effect of resveratrol on H2B expression (Fig. 7F). The above results, taken together, strongly support a notion that SIRT1 is a co-repressor for histone gene transcription.

### SIRT1 associates with and impacts histone acetylation status on histone promoters

**Figure 8:**
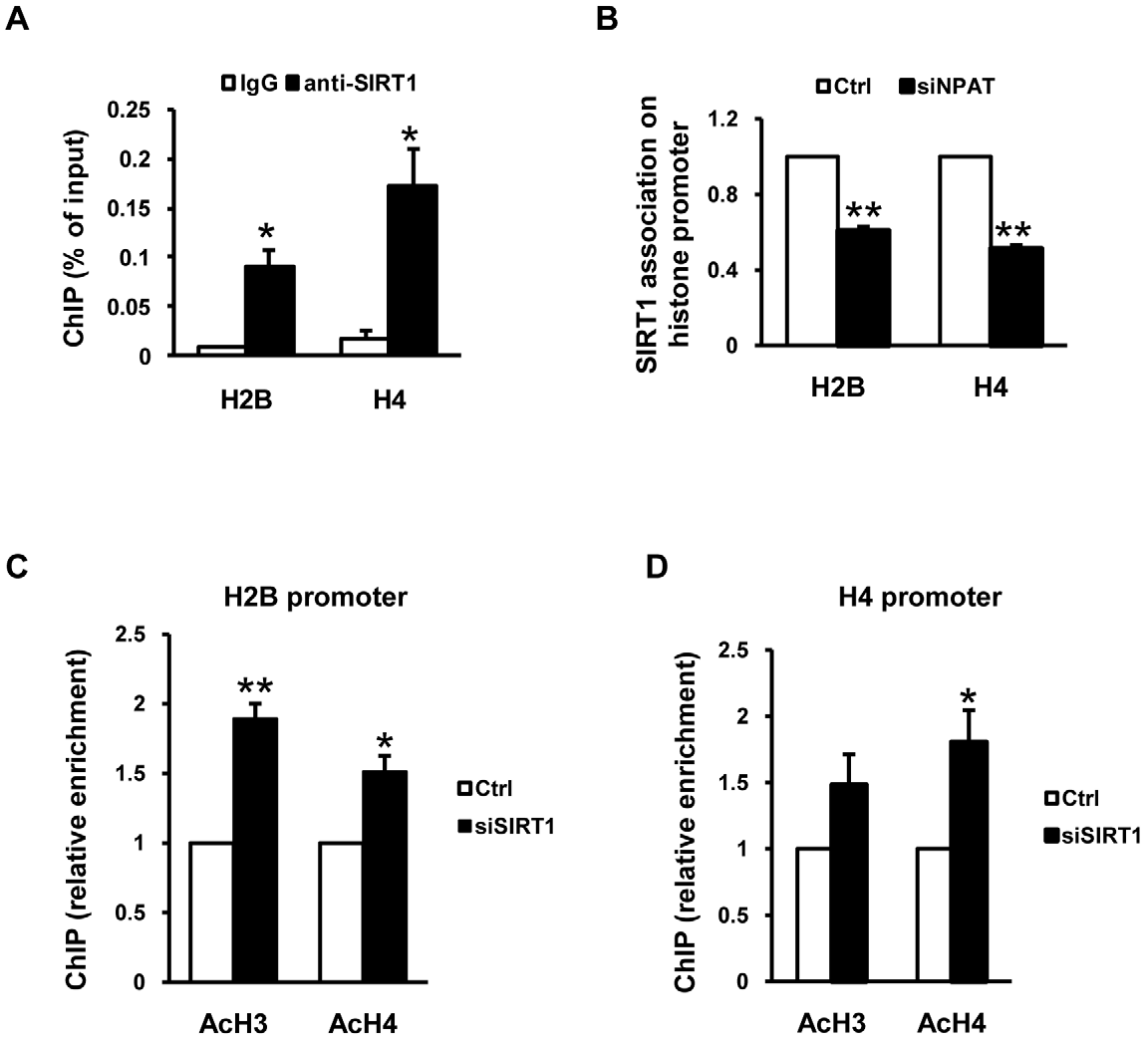
SIRT1 associates with histone gene promoters and impacts histone acetylation on target promoters. (A) SIRT1 associates with histone H2B and H4 promoters. (B) The SIRT1 association with histone promoters requires NPAT. (C) Increased levels of acetylated H3 and H4 on the H2B promoter in SIRT1 knock-down cells. (D) Increased levels of acetylated H3 and H4 on the H4 promoter in SIRT1 knock-down cells. In A–D, n = 3.

SIRT1 was previously shown to physically interact with p300 and Tip60 [56], [57]. As shown in Figure 4, CBP/p300 associates with histone promoters in an NPAT-dependent manner. In addition, it was reported that Tip60, another HAT complex, is also recruited to histone promoters by NPAT [58]. Thus, we reasoned that SIRT1 impacts histone gene transcription by associating with target promoters and performed ChIP assays; SIRT1 protein was indeed found to associate with the H2B and H4 promoters (Fig. 8A). In ChIP assays with NPAT knockdown cells, SIRT1 association with H2B and H4 promoters was 40–50% reduced (Fig. 8B), suggesting that SIRT1 is recruited to histone promoters in an NPAT-dependent manner. Consistent with the idea that histone-promoter-associated SIRT1 deacetylates histones, ChIP results showed that knockdown of SIRT1 increased the histone acetylation levels by 1.5 to 1.9 folds at the H2B and H4 promoters (Fig. 8C and 8D). These results suggest that SIRT1 associates with histone promoters, where it deacetylates histones to antagonize the functions of CBP/p300 and Tip60 to fine-tune histone expression during S-phase progression (also see Discussion).

### Growth phenotypes of CBP or SIRT1 knockdown cells

**Figure 9:**
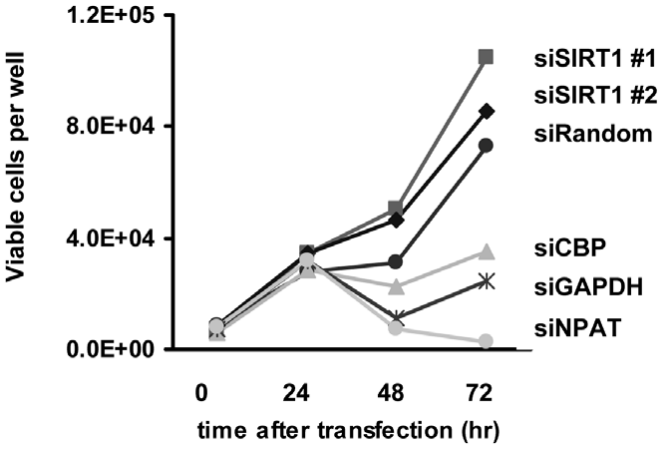
Growth phenotypes of HeLa cells knockdown of CBP or SIRT1. HeLa cells were transfected with 100 nM of different siRNA as indicated. At different time points post transfection, the numbers of viable cells were counted with tryphan blue staining. Random siRNA and siRNA specific for p38/GAPDH or NPAT were controls. Results were mean of triplicates.

Sustained histone mRNA expression defects should eventually feedback to histone protein levels to negatively impact DNA replication hence defects of cell proliferation as secondary effects of histone expression deficiency. This was prominently demonstrated in Drosophila cells [59]. We investigated the growth patterns of HeLa cells subjected to knockdowns of several histone expression regulators. SIRT1 knockdown resulted in a faster growth whereas the CBP knockdown led to a slower growth (Fig. 9). As additional validation, and given that p38/GAPDH (a component of OCA-S essential for H2B transcription) and NPAT (a global histone expression regulator) were previously shown to be crucial for histone expression [17], [19], we knocked down their expression and found slower growth of corresponding cells (Fig. 9). The opposing growth phenotypes of CBP- and SIRT1-knockdown cells are in agreement with their antagonizing functions in histone expression.

## Discussion

We and other researchers [58] identified different HATs, CBP, p300 and Tip60, regulating the acetylation of histones on histone gene promoters and their recruitment to histone promoters are all NPAT-dependent. Taken together, these results suggest that multiple HATs are involved in the regulating of histone promoter acetylation and they may work synergistically to achieve the rapid and efficient regulation of histone gene expression during cell proliferation and in response to environmental cues.

It was reported that histone promoters switch between relaxed status in S-phase when histone genes are transcribed and condensed status in G1 and G2/M phases when histone genes are not activated [60], [61]; however, the underlying mechanism(s) remained largely unknown. The impact of histone modifications on chromatin structure and gene activities has been extensively studied. The most notable histone modification has been the acetylation, which contributes to relaxed chromatin structure in two ways: first, it reduces the positive charges of histones and weakens the interactions between histones and negatively charged DNA; second, acetylated lysine residues in the N-terminal tails may generate interacting surfaces for bromo-domain containing chromatin remodeling activities such as the SWI/SNF complexes [9]. Here we have revealed that transcriptional co-activators CBP and its homolog p300 associate with histone promoters in an NPAT dependent manner to acetylate the H3 and H4 N-terminal tails, and are essential for optimal S-phase histone expression. The cell cycle regulated acetylation of histones catalyzed by CBP/p300, and potentially by Tip60 as well, on histone promoters might in combination contribute to oscillatory promoter structural changes in concert with oscillatory histone expression.

Histone gene expression and DNA replication is tightly coupled. When cell cycle progresses to the G1/S transition, cyclin E/cdk2 is part of a signaling cascade that leads to up-regulated expression of several proteins involved in DNA replication; meanwhile, cyclin E/cdk2 phosphorylates NPAT, a global regulator of core histone gene expression. The fact that both DNA replication and histone transcription are downstream of cyclin E/cdk2 somewhat explains the coupling of the two cell cycle events [19]–[21], [23]. Upon DNA damage, DNA replication is suspended and histone transcription is correspondingly stopped [62], [63]. The mechanism for the inhibition of histone transcription as a result of DNA damage is not well understood. Previous studies showed that DNA damage by irradiation treatment activates the ATM/ATR-p53-p21 pathway, and up-regulated p21 feeds back to inhibit cyclin E/cdk2 and results in repression of both DNA replication and histone transcription [62], [63]. However, other studies showed that cyclin E/cdk2 is only inhibited upon short DNA damage but not for prolonged DNA damage treatments, suggesting that the coupled inhibition of DNA replication and histone transcription under the latter condition was independent of cyclin E/cdk2 [3]. These observations indicate complicated mechanisms that couple DNA replication to histone expression.

In this study, we show that histone acetylases CBP and p300 associate with histone promoters and are important for histone gene activation. In addition to their role in histone gene regulation, CBP and p300 are known to participate in DNA damage repair. Firstly, CBP/p300 catalyzes the acetylation of histone H3 lysine 56 which is critical for chromatin assembly following DNA replication and repair [64]. Secondly, CBP/p300 physically interacts with and acetylates thymine DNA glycosylase which initiates DNA repair of G/T and G/U base mismatch [65], and 8-oxoguanine-DNA glycosylase 1 which is responsible for repair of mutagenic DNA lesion [66]. Thirdly, CBP/p300 physically interacts with proteins in DNA damage repair complexes, such as BRCA1, PCNA, ATR and DDB (damaged-DNA binding protein), which suggests direct involvement of CBP/p300 in DNA repair at DNA damage sites [67]–[69]. Similar to CBP/p300, Tip60 is another histone acetylase that functions in both histone gene activation and DNA damage repair [58], [70]–[72]. The involvement of CBP/p300 and Tip60 in these two tightly coupled events might suggest a coordinator role of these HATs, and the underlying mechanisms are subjects for further detailed investigation.

Our earlier studies have shown that the SSCS of histone H2B promoter is occupied by Oct-1 throughout the cell cycle which recruits OCA-S complex, an essential complex for histone H2B gene activation, in S-phase [17]. The direct interaction of Oct-1 with p38/GAPDH, the key subunit of OCA-S complex, is modulated by NAD(H) [17]. Similar to yeast metabolic cycle [73], the mammalian metabolic cycle (MMC) was implied by oscillation of NAD+/NADH ratios in a cell cycle [49]; in particular, the NAD+/NADH ratios are lower in the S phase [49] especially at G1/S transition and early S phases (data not shown); fluctuating NAD+/NADH ratios were found to heavily impact histone expression [48], [49]. Here, we found that the NAD+-dependent histone deacetylase, SIRT1, is associated with histone H2B and H4 promoters and represses histone H2B and H4 transcription. This novel observation adds another layer of complication for the link between histone expression and cellular metabolism and redox status.

**Figure 10:**
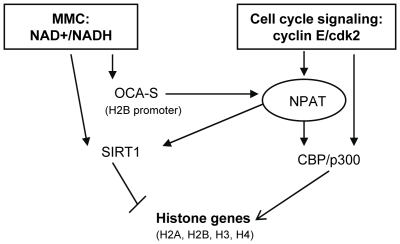
A model for the regulation of histone gene transcription. At G1/S transition of a cell cycle, the cellular NAD+/NADH ratio is proper for the assembly of the OCA-S complex which facilitates the recruitment of NPAT to the histone H2B promoter. NPAT, phosphorylated by cyclin E/cdk2, in turn facilitates the recruitment of CBP/p300 and SIRT1 to histone promoters in a global fashion. CBP/p300, which is also phosphorylated by cyclin E/cdk2, enhances histone acetylation on histone promoter regions hence activating S-phase histone transcription. In late S phase, the CBP/p300 association with histone promoters is decreased, likely accompanied by increased SIRT1 recruitment, which in conjunction with more oxidative cellular redox (higher NAD+ level) stimulates the HDAC activity of SIRT1 specifically targeting histone gene promoters hence accounting for declination of histone expression in a coordinated fashion. This model adds another layer of regulation of redox-modulated histone expression.

Since SIRT1 association with histone H2B and H4 promoters is mediated by NPAT, the global histone gene regulator, it is very likely that SIRT1 also associates with other histone promoters and plays a role in the coordinated regulation of different core histone genes in response to cellular redox status. Given that the HDAC activity of SIRT1 is NAD+-dependent, it could be correspondingly low in early S-phase progression hence allowing the effects of CBP/p300 and Tip60 on histone expression to be dominant. When cells progress into late S phase, however, MMC dictates that the cellular redox status becomes more oxidative. Under this circumstance, SIRT1 may be more potent because of a relatively higher NAD+ level, leading to histone deacetylation on the target promoters and repression of histone genes (Fig. 10).

In summary, we observed the association of HATs, CBP and p300, and HDAC, SIRT1, with histone promoters in an NPAT-dependent manner and their influences on histone promoter acetylation and histone gene transcription. These observations imply that the fluctuating expression of histone genes upon S-phase entry and during S-phase progression (e.g., Fig. 1B and Fig. 7A) may be a function of dynamic associations of diverse positive and negative transcription regulators that are under the control of the cyclinE/cdk2 signaling, in conjunction with certain feedbacks from cellular metabolism and redox status (Fig. 10).

## Materials and Methods

### Cell culture and treatments

HeLa S [17] and HEK293T [45] cells were cultured in Dulbecco's modified Eagle's medium (DMEM; Sigma) supplemented with 10% fetal bovine serum (HyClone), 1% Antibiotic/Antimycotic (Invitrogen) and 1 mM L-Glutamine (Invitrogen). G2/M phase synchronization of HeLa cells was achieved with nocodazole (75 ng/ml, 20 hours). G1/S phase synchronization was achieved with hydroxyurea (HU) (2.5 mM, 24 hours). Chemicals for cell treatments, including nocodazole, HU, TSA (trichostatin A), NAM (nicotinamide) and resveratrol, were purchased from Sigma; the SIRT1 inhibitor III was purchased from Calbiochem.

### Cell transfection

For RNA interference, HeLa S cells at 30% confluency were transfected with 100 nM of siRNA using Lipofectamine RNAiMAX (Invitrogen) followed the manufacturer's instruction. Cells were harvested for analysis at 48 hours after transfection. Sequences of siRNA used in this study were listed below. Random siRNA: UCAGUGUCAUACGUACGACGdTdT. siCBP: GCAAGAAUGCCAAGAAGAAdTdT. sip300: AGGAGGAAGAAGAGAGAAAdTdT. siNPAT: GGGUUUGCGAAGUGAGAAAdTdT. siSIRT1#1: GAUGAAGUUGACCUC CUCAdTdT. siSIRT1#2: GGAAAUAUAUCCUGGACAA-dTdT. siSIRT1#1 was used in Figures 7, 8 and 9 unless otherwise indicated. siGAPDH: GUGUGAACCAUGAGAAGUAdTdT.

For p300 overexpression, HeLa S or HEK293T cells at 90% confluency were transfected with pCMVb p300 HA (Addgene plasmid 10718; note: it was recently notified by Addgene that the p300 protein encoded by this plasmid was missing one amino acid, N2379, near the very C-terminus. N2379 is not conserved between p300 and CBP and is not within identified protein-interacting or functional domains of p300 [27], thus missing this amino acid would not affect the functions of p300 in our assay systems), or pcDNA3.1-p300 (HAT-) (Addgene plasmid 23254) plasmids using Lipofectamine 2000 (Invitrogen) as suggested by the manufacturer. Cells were harvested for analysis 48 hours after transfection.

### Extraction of total RNA and RT-qPCR

Total RNA was extracted with RNeasy mini Kit (Qiagen). Reverse transcription was performed with SuperScript III reverse transcriptase kit (Invitrogen). Complementary DNA was quantified by real-time qPCR using KAPA SYBR FAST qPCR MasterMix kit (Kapabiosystems) and the 7300 Real-Time PCR system (Applied Biosystems). Primers used in qPCR reactions were: cyclin E-forward: CGTGCGTTTGCTTTTACAGA; cyclin E-reverse: AGCACCTTCCATAGCAGCAT. Primers for H2B, H4 and β-actin were as described [49].

### Chromatin immunoprecipitation (ChIP)

For ChIP assays with anti-AcH3 (Upstate, #06-599), anti-AcH4 (Upstate, #06-598, #06-866), anti-RNA polymerase II (Covance, #MMS-126R) or anti-NPAT (anti-NPAT antibodies were raised in rabbits using a bacterially expressed NPAT fragment, and further purified with protein A-conjugated Sepharose beads) antibodies, single cross-linking was performed. HeLa cells were treated with 1% of formaldehyde (Sigma) for 15 minutes at room temperature with gentle shaking. For ChIP assays with anti-CBP (Santa Cruz, sc-369), anti-p300 (Santa Cruz, sc-585) or anti-SIRT1 (Upstate, #04-1091) antibodies, double cross-linking was performed. HeLa cells were firstly treated with 10 mM of disuccinimidyl glutarate (DSG) for 45 minutes to crosslink proteins and subsequently treated with 1% formaldehyde for 15 minutes to crosslink proteins with DNA. After cross-linking, cells were collected and washed three times with MC buffer (10 mM of Tris.Cl pH7.5, 10 mM of NaCl, 3 mM of MgCl2, 0.5% of NP40). Chromatin DNA was fragmented with sonication to the average size of 200–500 bp. Protein A Agarose beads (upstate, #16-157) were used to precipitate chromatin DNA complexes with indicated antibodies. Procedures for ChIP assays and primers for H2B and H4 promoters were as described [17].

### Fluorescence activated cell sorting (FACS)

HeLa cells were harvested, washed with PBS, fixed with 80% cold ethanol and left at 4°C for >30 minutes. Afterwards, cells were washed twice with PBS and incubated in 200 µl of propidium iodide (50 µg/ml, Sigma) containing RNase A (20 µg/ml, Sigma) for 30 minutes in dark, before being analyzed by flow cytometry (BD company).

### Coimmunoprecipitation (co-IP) and Western-blot

HeLa cells were transfected with pCMV-NPAT plasmids (a kind gift from Dr. Jiyong Zhao, University of Rochester Medical Center, US). Cells were harvested 24 hours posttransfection and whole cell extracts were prepared with RIPA buffer (50 mM Tris-HCl, pH 7.4, 150 mM NaCl, 0.25% deoxycholic acid, 1% NP-40, 1 mM EDTA, 1 mM NaF). Whole cell extracts containing 1 mg of total proteins were incubated with 20 µl of rabbit anti-CBP (Santa Cruz, sc-369) in binding buffer [50 mM Tris-HCl, pH 7.4, 150 mM NaCl, 0.25% deoxycholic acid, 1% NP-40, 1 mM EDTA, 1 mM NaF, 1× protease inhibitors (cOmplete, EDTA-free, tablets, Roche)] at 4°C overnight and 10 µl of Dynabeads protein A (Invitrogen) were added for another 1 hour. Beads were washed 3 times with binding buffer. Co-IP products and 2% of starting material for co-IP were loaded for SDS-PAGE (5% gel). Proteins were transferred to nitrocellulose membrane (Hybond-C Extra, Amersham Bioscience) in 12.5 mM CAPS, pH 10.8 buffer and the membrane was blotted with mouse anti-CBP (Santa Cruz, sc-7300) or mouse anti-NPAT (BD Biosciences, Cat:611344). The bound primary antibodies were detected with HRP-conjugated goat anti-mouse IgG (Amersham Bioscience) and visualized with ECL plus kit (GE Healthcare).

Primary antibodies used in Western-blots include anti-CBP (Santa Cruz, sc-7300), anti-p300 (Santa Cruz, sc-585), anti-Sti1 (raised in rabbits with bacterially expressed recombinant protein), anti-GAPDH (Chemicon, MAB374), anti-SIRT1 (Santa Cruz, sc-15404), anti-tubulin (invitrogen, 23610501), anti-NPAT (raised in rabbit or purchased from BD Biosciences), anti-Acetyl-p53 (cell signaling, #2525), anti-p53 (Santa Cruz, sc-126), anti-HA (Santa Cruz, sc-805).

### Promoter activity assay

Promoter activity assay was carried out as previously described [17]. HeLa cells were transfected with firefly luciferase reporter plasmids (driven by histone H2B promoter) and renilla luciferease control plasmids (driven by the thymidine kinase promoter). At 24 hours, cells were treated with 50 nM SIRT1 inhibitor III for additional 24 hours. Firefly or renilla luciferase activity was measured with the Dual-Luciferase Reporter Assay System (Promega) using a TD-20/20 luminometer (Turner Biosystems).

### Statistical analyses

Data were analyzed with GraphPad Prism program. In Figure 1, data collected in G2/M phase were set as 1 and data collected in G1, G1/S and S phase G2/M phases were normalized to G2/M group and analyzed with one-sample *t* test with the hypothetical value as 1. In Figures 2, 3, 4E, 4F, 6, 7 and 8B–D, data of experimental groups were normalized to the control groups and analyzed with one-sample *t* test with the hypothetical value as 1 or 100. In Figures 4A, 4B and 8A, data of specific antibody group(s) were compared with normal IgG group and analyzed with unpaired *t* test. In Figure 5A, data of the G1, G1/S or S groups were compared with the G2/M group, respectively, and analyzed with unpaired *t* test. Two tailed *p* value<0.05 or <0.01 was indicated by * or **, respectively. Data were presented as Mean + SEM.
